# Prevalence of Potentially Inappropriate Apixaban Prescribing Within a Single‐Centre Tertiary Hospital

**DOI:** 10.1111/ajag.70166

**Published:** 2026-04-17

**Authors:** Katherine J. Creeper, Grace Lenaghan, Aaron Stockwell, Matthew Schmidt, Katharine Ingram, Andrew C. Stafford

**Affiliations:** ^1^ Haematology Department Sir Charles Gairdner Hospital Nedlands Western Australia Australia; ^2^ Haematology Department PathWest Laboratory Medicine Nedlands Western Australia Australia; ^3^ Curtin Medical School Curtin University Bentley Western Australia Australia; ^4^ Pharmacy Department Sir Charles Gairdner Hospital Nedlands Western Australia Australia; ^5^ Department of Geriatric Acute and Rehabilitation Medicine Sir Charles Gairdner Hospital Nedlands Western Australia Australia

**Keywords:** aged, anticoagulants, atrial fibrillation, drug therapy, venous thromboembolism

## Abstract

**Objective:**

Apixaban is widely recommended as first line therapy for atrial fibrillation (AF) and venous thromboembolism (VTE). Despite the widespread availability of dosing guidelines, anecdotal evidence suggests clinicians do not routinely follow these recommendations. Inappropriate dose reduction is associated with suboptimal patient outcomes. This project aimed to review current prescribing practices of apixaban 2.5 mg against guidelines to identify potentially inappropriate prescribing.

**Method:**

A single‐centre, retrospective review of all adult patients admitted to a large tertiary hospital in Perth, Australia, who were dispensed apixaban 2.5 mg between 1 January 2023 and 31 December 2024, was undertaken. Patients were identified via dispensing records. Demographics, indication, comorbidities, concurrent medications and laboratory data were collated. Each case was assessed against current guidelines to determine whether dosing was appropriate or potentially inappropriate. Data were analysed using quantitative statistics.

**Results:**

A total of 303 patients were dispensed apixaban 2.5 mg in the audit period. Of these 178 (59%) were newly initiated. The mean age of the cohort was 79 years (standard deviation [SD] 12.6), with 49% female. Of the 128 patients (72%) newly initiated on apixaban 2.5 mg for non‐valvular AF, 53 (41%) patients received a potentially inappropriate dose. A further 19 patients (11% of the entire cohort) received a potentially inappropriately low dose for acute VTE treatment.

**Conclusions:**

A significant proportion of patients initiated on apixaban 2.5 mg received potentially inappropriate doses. These findings highlight the need for targeted anticoagulation stewardship and clinician education to optimise patient outcomes.

## Practice Impact

This study highlights the significant proportion of patients who receive discordant doses of anticoagulation to national guidelines. The lack of a clear reason suggests multiple factors are incorporated into risk assessment models and further research on the clinical implications of these decisions is needed.

## 1 Introduction

Direct oral anticoagulants (DOACs), such as apixaban, have become first line agents in the management of non‐valvular atrial fibrillation (AF) and venous thromboembolism (VTE). Their predictable pharmacokinetics, fixed dosing regimens and favourable safety profile, particularly with regard to reduced rates of intracranial haemorrhage, make them preferable to vitamin K antagonists (VKAs), such as warfarin [1]. Furthermore, DOACs offer the practical advantage of not requiring routine therapeutic monitoring, thereby simplifying long term anticoagulation for both patients and clinicians [2]. For all indications, clinical guidelines recommend fixed DOAC doses, with dose adjustments only recommended under specific circumstances. These include the presence of specific clinical conditions (e.g., renal function), patient characteristics (e.g., age, body weight) and concomitantly administration with other drugs that alter DOAC pharmacokinetics (e.g., modulators of P‐glycoprotein and/or of cytochrome P450 isoenzymes) [3].

Atrial fibrillation is particularly common in older patients, with more than 10% of people older than 80 years of age requiring anticoagulation for secondary stroke prevention [4]. Similarly, advancing age is strongly associated with increased risk of bleeding [5]. The competing risks of advancing age and increased bleeding tendency may, however, be less of a concern with apixaban versus warfarin due to supportive data demonstrating improved efficacy with fewer bleeding complications [6]. The improved safety and tolerability profile of DOACs have correlated to a substantial increase in anticoagulant use and expenditure. Primarily, this is attributable to an increased number of patients being initiated on oral anticoagulants and a lesser proportion of patients transitioning from vitamin K antagonists to DOAC [7]. Nevertheless, there likely remains a residual proportion of prescribers or patients that are cautious about their use.

Australian guidelines for apixaban recommend a standard dose of 5 mg twice daily for stroke prevention in non‐valvular AF. A reduced dose of 2.5 mg twice daily is only recommended for patients who meet at least two of the following criteria: age 80 years or older, body weight ≤ 60 kg or serum creatinine ≥ 133 μmol/L [3]. In the treatment of acute VTE, the initial recommended dose is 10 mg twice daily for 7 days, followed by 5 mg twice daily. After a minimum of 6 months of therapy, a reduced maintenance dose of 2.5 mg twice daily may be considered for extended secondary prevention [3].

Despite these clearly defined dosing criteria, international real‐world studies have consistently demonstrated suboptimal adherence to DOAC dosing guidelines, particularly in older populations where clinicians are particularly concerned about bleeding. However, inappropriately reduced dosing may result in subtherapeutic anticoagulation, thereby increasing the risk of stroke or recurrent thromboembolism. Multiple studies have reported that both under‐ and overdosing of DOACS is associated with significantly worse clinical outcomes, including higher rates of cardiovascular events, hospitalisation and mortality, with no significant reduction in bleeding [8, 9].

There is a paucity of data evaluating the appropriateness of apixaban 2.5 mg dosing initiated in adults admitted to Australian healthcare facilities. The aim of this study was to evaluate the prevalence of potentially inappropriate apixaban 2.5 mg prescribing in an Australian tertiary hospital setting. We focused our analysis exclusively on the 2.5 mg dose as underdosing is a well‐recognised issue in international studies.

## 2 Methods

### 2.1 Study Design and Setting

A single‐centre retrospective observational cohort study of all adult patients admitted to a tertiary hospital in Western Australia between 1 January 2023 to 31 December 2024 was conducted. Ethics approval was granted by the local quality improvement hospital committee and data extraction was performed in compliance with institutional governance protocols.

### 2.2 Inclusion Criteria

All adult patients (aged 18 years and older) who were admitted to hospital and were newly initiated on and dispensed apixaban 2.5 mg during the study period were included.

### 2.3 Data Collection

Patients were initially identified by retrospectively interrogating hospital pharmacy dispensing software. Patient medical records were reviewed and baseline demographics, comorbidities, concurrent medications, indication for anticoagulation and haematological and biochemical laboratory parameters were collated and recorded. Covariates were selected based on clinical relevance in addition to variables incorporated in CHA_2_DS_2_‐Va and HAS‐BLED risk assessments [10, 11]. A history of major bleeding was documented and classified in accordance with the International Society on Thrombosis and Haemostasis (ISTH) criteria (Table S1) [12]. Data were collated in a standardised Excel spreadsheet (Microsoft, Redmond, Washington, USA) with predefined columns and drop‐down options. Following data collection, HAS‐BLED scores were calculated for all patients, and CHA_2_DS_2_‐Va scores were calculated for all patients newly initiated on apixaban for AF [11, 12].

### 2.4 Dosing Appropriateness Assessment

For patients newly dispensed apixaban 2.5 mg, the potential appropriateness of the dose was evaluated according to Australian and relevant international guidelines as follows:

- For non‐valvular AF, a reduced dose of 2.5 mg twice daily was classified as appropriate if:
  ○ The patient met at least two of the following criteria: age 80 years or older, body weight ≤ 60 kg or serum creatinine ≥ 133 μmol/L, or
  ○ The patient's eGFR was < 30 mL/min/1.73 m^2^
- For VTE treatment, 2.5 mg twice daily was only considered appropriate when used for extended secondary prophylaxis after a minimum of 6 months' treatment at a higher dose

Prescriptions deviating from these dosing recommendations without meeting the qualifying criteria were classified as potentially inappropriate dosing.

### 2.5 Statistical Analysis

Demographic characteristics were summarised using descriptive statistics. Continuous parametric variables were reported as means with standard deviations (SD). Continuous non‐parametric variables were reported as medians and interquartile range (IQR). Categorical variables were presented as frequencies and percentages. Group comparisons between appropriate and potentially inappropriate dosing were conducted using Student's *t*‐tests for continuous variables and *χ* ^2^ tests for categorical variables.

To identify independent predictors of potentially inappropriate apixaban dosing, we fit a binary logistic regression model (dependent variable: potentially inappropriate apixaban dosing vs. appropriate dosing), reporting odds ratios (ORs) and 95% confidence intervals (CIs). The predictors included in the model were all factors found to be predictors of potentially inappropriate apixaban dosing in univariate analyses, excluding the HAS‐BLED score as the parameters used in its calculation were already evaluated in univariate analyses. Variables were entered into the regression models using the forward stepwise method. Variable entry and removal thresholds were set at *p* < 0.05 and *p* > 0.10, respectively. The Nagelkerke *R* ^2^ is reported as a measure of explained variance.

All analyses were performed using IBM SPSS Statistics version 31 (IBM, New York, USA). For all analyses, a two tailed *p* value < 0.05 was considered statistically significant.

## 3 Results

### 3.1 Patient Demographics

A total of 303 patients were dispensed apixaban 2.5 mg during the study period. The mean age was 79.2 years (SD 12.6) and 149 patients (49%) were female. The mean body weight of the cohort was 73.2 kg (SD 19.8), equating to a mean body mass index (BMI) of 26.5 kg/m^2^ (SD 5.9). Of the medical conditions for which data were collected, the most prevalent comorbidities in the cohort were cardiovascular conditions, including hypertension (70%, *n* = 213), congestive cardiac failure (38%, *n* = 116) and ischaemic heart disease (37%, *n* = 37). Active malignancy was present in 56 (19%) of patients, whereas 66 (22%) had previously experienced a stroke or transient ischemic attack (TIA). Sixty‐one patients (20%) had a history of major bleeding and 94 (31%) had a history of falls within the preceding 12 months. Regarding concurrent medications, 94 patients (31%) were concomitantly prescribed antiplatelet therapy. The mean HAS‐BLED score for the cohort was 2.7 (SD 1.2), with males exhibiting a slightly higher average bleeding risk compared with females (2.9 vs. 2.4) (Table 1).

**TABLE 1:** Demographics of the entire cohort.

Characteristic	Female (*n* = 149, 49% of total)	Male (*n* = 152, 50% of total)	Total (*n* = 303)^b^
Age years (mean, std. dev)	80.3 (13.4)	78.3 (11.7)	79.2 (12.6)
Weight kg (mean, std. dev)	65.0 (19.0)	81.8 (16.8)	73.4 (19.8)
BMI kg/m^2^ (mean, std. dev)	25.7 (6.5)	27.3 (5.1)	26.5 (5.9)
Comorbidities (*n*, % of column)			
Hypertension	99 (66)	114 (75)	213 (70)
Congestive cardiac failure	52 (35)	64 (42)	116 (38)
Ischaemic heart disease	31 (21)	51 (34)	82 (27)
Diabetes	40 (27)	67 (44)	107 (35)
Stroke/transient ischemic attack	34 (23)	31 (20)	66 (22)
Peripheral vascular disease	7 (5)	16 (11)	23 (8)
Active cancer	27 (18)	28 (18)	56 (19)
History of bleeding^a^	26 (17)	34 (22)	61 (20)
History of falls in preceding 12 months	53 (36)	41 (27)	94 (31)
Concurrent antiplatelet use (*n*, % of column)			
Aspirin	25 (17)	38 (25)	63 (21)
Clopidogrel	6 (4)	14 (9)	20 (7)
Dual antiplatelet	1 (1)	10 (7)	11 (4)
Total	32 (22)	62 (41)	94 (31)
HAS‐BLED score (mean, std. dev)	2.4 (1.2)	2.9 (1.2)	2.7 (1.2)

Abbreviations: BMI, body mass index; std. dev, standard deviation; VTE, venous thromboembolism.

^a^ Any history of bleeding requiring hospitalisation and/or causing a decrease in haemoglobin level of > 20 g/L and/or requiring blood transfusion that was not a haemorrhagic stroke.

^b^ Includes data for two transgender patients.

### 3.2 Indication for Anticoagulation

Of the 303 patients, apixaban 2.5 mg was newly initiated in 178 (59% of the cohort), of which 84 were female (49% of all females in the cohort). Atrial fibrillation was the primary indication for initiation of apixaban 2.5 mg, accounting for 76% of all patients (*n* = 230, Table 2). Of the patients dispensed apixaban 2.5 mg for VTE, 28 (9% of the cohort) were for prophylaxis and 19 (6%) were for acute treatment.

**TABLE 2:** Characteristics of apixaban 2.5 mg prescribing.

Characteristic	Female (*n* = 149, 49% of total)	Male (*n* = 152, 50% of total)	Total (*n* = 303)^a^
Newly prescribed (*n*, % of column)	84 (56)	92 (61)	178 (59)
Indication for commencement of anticoagulation (*n*, % of column)			
AF	114 (77)	116 (76)	230 (76)
Acute VTE	8 (5)	11 (7)	19 (6)
Prophylactic VTE	16 (11)	11 (7)	28 (9)
SVT	6 (4)	3 (2)	10 (3)
Other	5 (3)	11 (7)	16 (5)

Abbreviations: AF, Atrial fibrillation; SVT, superficial vein thrombosis; VTE, venous thromboembolism.

^a^ Includes data for two transgender patients.

### 3.3 Appropriateness of Apixaban Dosing

Of the 128 patients who were initiated on apixaban 2.5 mg for AF, 53 (41%) received a potentially inappropriate dose based on the study criteria (Table 3). Patients in the potentially inappropriate dosing group had significantly higher body weight (mean 76.8 kg vs. 68.1 kg; *p* = 0.02) and a lower proportion were diagnosed with hypertension (82.7% vs. 66.0%; *p* = 0.03) than the remainder of the cohort. Although there was no significant difference between the two groups' CHA_2_DS_2_‐Va scores, the mean HAS‐BLED score was significantly lower in the potentially inappropriate dosing group (2.6 vs. 3.0; *p* = 0.049).

**TABLE 3:** Comparisons between appropriate and potentially inappropriate patients commencing anticoagulation with apixaban 2.5 mg for atrial fibrillation (univariate analysis).

Characteristic	Appropriate (*n* = 75, 59% of total)	Potentially inappropriate (*n* = 53, 41% of total)	Total cohort (*n* = 128)	Unadjusted odds ratio (95% confidence interval)
Age years (mean, std. dev)	82.5 (9.8)	79.2 (9.6)	81.2 (9.8)	0.965 (0.930 to 1.002), *p =* 0.07
Weight kg (mean, std. dev)	68.1 (19.5)	76.8 (18.2)	71.6 (19.4)	1.024 (1.004 to 1.044), *p =* 0.02*
BMI kg/m^2^ (mean, std. dev)	25.4 (5.4)	27.2 (6.5)	26.2 (5.9)	1.054 (0.984 to 1.130), *p =* 0.14
Female sex (*n*, % of column)	41 (55)	22 (42)	63 (49)	1.699 (0.835 to 3.459), *p =* 0.14
Comorbidities (*n*, % of column)				
Hypertension	62 (83)	35 (66)	97 (76)	0.408 (0.179 to 0.930), *p =* 0.03*
Congestive cardiac failure	39 (52)	21 (40)	60 (47)	0.606 (0.297 to 1.236), *p =* 0.17
Ischaemic heart disease	26 (35)	11 (21)	37 (29)	0.494 (0.218 to 1.117), *p =* 0.09
Diabetes	23 (31)	22 (42)	45 (35)	1.604 (0.770 to 3.344), *p =* 0.21
Stroke/transient ischemic attack	16 (21)	15 (28)	31 (24)	1.456 (0.645 to 3.285), *p =* 0.37
Peripheral vascular disease	6 (8)	2 (4)	8 (6)	0.451 (0.087 to 2.327), *p =* 0.34
Active cancer	8 (11)	8 (15)	16 (13)	1.489 (0.521 to 4.256), *p =* 0.46
History of bleeding^a^	15 (20)	18 (34)	33 (26)	0.486 (0.218 to 1.084), *p =* 0.08
History of falls in preceding 12 months	23 (31)	22 (42)	45 (35)	0.623 (0.299 to 1.299), *p =* 0.21
Concurrent antiplatelet use (*n*, % of column)				
Aspirin	14 (19)	13 (25)	27 (21)	1.305 (0.550 to 3.098), *p =* 0.55
Clopidogrel	6 (8)	2 (4)	8 (6)	0.468 (0.090 to 2.451), *p =* 0.37
Dual antiplatelet	3 (4)	1 (2)	4 (3)	0.468 (0.047 to 4.682), *p =* 0.52
Total	23 (31)	16 (30)	39 (31)	0.978 (0.455 to 2.100), *p =* 0.95
HAS‐BLED score (mean, std. dev)	3.0 (1.1)	2.6 (1.2)	2.9 (1.1)	0.725 (0.523 to 1.000), *p =* 0.049*
HAS‐BLED components not stated above (*n*, % of column)				
Renal disease	24 (32)	0 (0)	24 (19)	n/a
Liver disease	5 (7)	3 (6)	8 (6)	1.190 (0.272 to 5.212), *p* = 0.82
Age > 65 years	72 (96)	48 (91)	120 (94)	2.500 (0.571 to 10.952), *p* = 0.22
Medication usage predisposing to bleeding	26 (35)	19 (36)	45 (35)	0.950 (0.455 to 1.982), *p* = 0.89
Alcohol use	7 (9)	1 (1.9)	8 (6.3)	5.353 (0.639 to 44.872), *p* = 0.12
CHA_2_DS_2_Va score (mean, std. dev)	4.2 (1.4)	3.8 (1.8)	4.0 (1.6)	0.842 (0.668 to 1.061), *p =* 0.14

Abbreviations: BMI, body mass index; std. dev, standard deviation; VTE, venous thromboembolism.

^a^ Any history of bleeding requiring hospitalisation and/or causing a decrease in haemoglobin level of > 20 g/L and/or requiring blood transfusion that was not a haemorrhagic stroke.

* Significant at *p* < 0.05.

Univariate analysis (Table 3) demonstrated that higher body weight and a diagnosis of hypertension were significantly associated with potentially inappropriate apixaban dosing. Both variables were included in a binary logistic regression model, which identified that only body weight was an independent predictor of dosing appropriateness, with increasing weight increasing the likelihood of potentially inappropriate prescribing (adjusted OR 1.03 [95% CI 1.00–1.05], *p =* 0.01, Table 4). The model explained 10% of the variance in potentially inappropriate apixaban dosing (Nagelkerke *R* ^2^ = 0.101).

**TABLE 4:** Binary logistic regression investigating hypertension and body weight as predictors of potentially inappropriate apixaban 2.5 mg dosing for atrial fibrillation.

Characteristic	Adjusted odds ratio (95% confidence interval)
Hypertension	0.421 (0.174–1.016) *p* = 0.05
Weight (kg)	1.027 (1.006–1.047) *p* = 0.01*

* Significant at *p* < 0.05.

Nineteen patients (6% of the cohort) were dispensed apixaban 2.5 mg for acute treatment of VTE. As the study criteria did not consider this dose to be appropriate under any circumstance, this dosing was potentially inappropriate for all these patients. To investigate the potential for the lower dose being dispensed due to prescribers applying the AF dosing criteria to these patients, the prevalence of the AF dose reduction criteria was investigated in this cohort. Eleven of the 19 patients (58%) would have met the criteria for dose reduction, had the indication been AF and not acute VTE.

## 4 Discussion

This study reviewed local prescribing practices for apixaban 2.5 mg and specifically adherence to dosage guidelines within hospitalised adult patients. Approximately 40% of new prescriptions for AF and 100% of prescriptions for acute VTE were potentially discordant with national guidelines. Although higher body weight and no diagnosis of hypertension were significantly associated with potentially inappropriate apixaban dosing, it remains unclear as to the clinical justification for the underdosing as neither CHA_2_DS_2_‐Va nor HAS‐BLED incorporate body weight into their risk prediction scores.

Results of previous similar studies highlight that these findings of potentially inappropriate anticoagulation prescribing are not unique to our institution. A cross‐sectional Australian study found that 43% of inpatients who were prescribed oral anticoagulants discordant with prescribing recommendations with 9% of these patients receiving a lower dose [13]. Another study by Ashraf et al. retrospectively reviewed 8125 patients with a diagnosis of AF or atrial flutter who commenced anticoagulation with a DOAC [14]. They reported that 21% of patients were inappropriately dosed, with the vast majority (98%) being underdosed. A subgroup analysis of the apixaban 2.5 mg cohort revealed that underdosing was associated with an increased incidence of all‐cause mortality (hazard ratio [HR] 1.24, 95% CI 1.03–1.49) [14]. A similar prevalence of apixaban underdosing was reported in a study by Decaix et al. [4].

Although the current study identified a higher prevalence of potentially inappropriate apixaban dosing than either Ashraf et al. [14] or Decaix et al. [4], other studies have reported higher rates than ours. A multicentre analysis by Harrington et al. [15] found that 61% (*n* = 4321) of patients who were prescribed apixaban 2.5 mg did not meet criteria for dose reduction. As with the study by Ashraf et al., Harrington et al. [15] reported that underdosing of apixaban was associated with higher rates of cardioembolic stroke, major bleeding and all‐cause death [15]. Although our study did not assess clinical outcomes, it is feasible that the potential underdosing of apixaban exposed the patients in our study to an increased risk of adverse outcomes without a clear benefit.

Although similar studies have reported a higher bleeding risk (HAS‐BLED score ≥ 3) as a significant predictor for incorrect prescribing [13], this was not the case in our analysis. In our study, diagnoses of hypertension and body weight were associated with inappropriate prescribing; however, we did not directly investigate the clinical reasons as to why a lower dose was initiated. Other studies provide some insight into the potential factors that influenced the prescribing decisions in our study. Our findings partially mirror those reported by Decaix et al. [4], who reported that cognitive impairment (OR 1.67, 95% CI 1.16–2.45, *p* < 0.05), weight per kilogram increase (OR 1.03, 95% CI 1.01–1.04, *p* < 0.001) and history of bleeding whilst taking apixaban or rivaroxaban (OR 1.94, 95% CI 1.24–3.03, *p* = 0.003) were associated with underdosing [4]. We therefore hypothesise that clinicians in our study may have incorporated other variables (which may not have been assessed), such as perceived frailty, fall risk, cognitive impairment, nutritional status as well as patient choice into their risk assessment, and reduced the dose accordingly. Despite the best doctor‐led discussions, older patients can decide against guideline‐aligned care.

Despite these considerations, guidelines advocate against withholding or reducing anticoagulant doses solely on the basis of age or fall risk. The 2021 UK National Institute for Health and Care Excellence (NICE) guidelines explicitly state that anticoagulation should not be withheld solely due to older age or a history of falls [16]. Likewise, the European Society of Cardiology (ESC) guidelines emphasise that a history of falls is not an independent predictor of bleeding with oral anticoagulants, and the benefits of anticoagulation typically outweigh the potential risks in older adults [17]. Concerns regarding the increased bleeding risk associated with both falls and advanced age may be overstated [18, 19]. One modelling study estimated that a patient would need to fall approximately 458 times per year for the bleeding risk of anticoagulation to outweigh its stroke prevention benefits [9]. Similarly, advancing age is also associated with increased risk of thromboembolic events. Thus, overly cautious dose reductions based on these concerns may paradoxically expose patients to avoidable harm.

Despite the high prevalence of potentially inappropriate apixaban dosing in this study, limitations related to the study design mean that its results should be interpreted with caution. As this study was conducted within a single institution and limited to hospitalised patients, the results may not be generalisable to other populations, such as outpatients. It is also unknown whether the doses of apixaban were chosen based on other patient factors (e.g., recent surgery), which were not captured by the data tool, or if the dose was revised during the admission or once the patients were discharged from hospital. Furthermore, as this study did not correlate clinical outcomes with potentially inappropriate dosing, the implications of our results remain unknown.

Another limitation is that our dataset did also not allow for detailed exploration of pharmacokinetic interactions or concurrent use of CYP3A4/P‐glycoprotein inhibitors that may have influenced prescribing practises. Although the extent of the interaction remains unknown, apixaban labelling recommends avoiding concomitant use of P‐glycoprotein and strong CYP3A4 inducers (e.g., rifampicin, carbamazepine and phenytoin). However, as no data currently exist to inform decision‐making based on drug levels, clinical outcomes and bleeding risk when these drug interactions are present [20], reduced dosing of apixaban in these circumstances may well have also been inappropriate.

These findings highlight a clear need for improved anticoagulation stewardship and clinician education to support guideline‐aligned dosing of apixaban. The high rate of potentially inappropriate dose reduction suggests that clearer local protocols and more consistent access to dosing guidance may help reduce variation in practice. Further research should explore the reasons clinicians choose reduced doses, including their interpretation of guidelines, and examine the clinical impact of inappropriate underdosing.

## 5 Conclusions

In this retrospective cohort study, a substantial proportion of hospitalised patients were dispensed a potentially inappropriate reduced dose of apixaban. The absence of clear independent predictors for dosing decisions underscores the complexity of anticoagulant prescribing in clinical practice. Efforts to enhance guideline adherence and incorporate comprehensive patient assessments may improve anticoagulant use and patient outcomes.

## Funding

This study was funded by the Charlies Leukaemia and Lymphoma Fund.

## Ethics Statement

Ethics approval was granted by the local quality improvement committee (activity number: 56390).

## Conflicts of Interest

The authors declare no conflicts of interest.

## Supporting information


**Table S1:** ajag70166‐sup‐000‐TableS1.docx.

## Data Availability

The data that support the findings of this study are available from the corresponding author upon reasonable request.
