# Supplementary material for: Prevalence of Potentially Inappropriate Apixaban Prescribing Within a Single‐Centre Tertiary Hospital
Source: Australas J Ageing. 2026 Apr 17;45:e70166. doi: 10.1111/ajag.70166 (PMC13087822; doi:10.1111/ajag.70166)
Supplement: Supplementary file 1 — Table S1: ajag70166‐sup‐000‐TableS1.docx. [file AJAG-45-0-s001.docx]

**Supplementary Table 1**

| **Demographics** | |
| --- | --- |
| - Age - Sex - Body weight | - Height - Body mass index (BMI) |
| **Clinical parameters** | |
| - Indication for anticoagulation - History of falls within the preceding 12 months | - Prior bleeding events^*^ |
| **Medical history** | |
| - Congestive heart failure (CCF) - Hypertension - Ischaemic heart disease - Stroke and/or transient ischaemic attack (TIA) | - Diabetes mellitus - Peripheral artery disease (PAD) - Active malignancy - Excessive alcohol consumption^†^ |
| **Laboratory data** | |
| - Haemoglobin - Platelet count - Serum creatinine - Estimated glomerular filtration rate (eGFR) | - Aspartate transferase (AST) - Alanine transaminase (ALT) - Bilirubin |
| **Co-administered medications** | |
| - Antiplatelet agents^‡^ | - Non-steroidal anti-inflammatory drugs (NSAIDs) |
| ^*^Any history of bleeding requiring hospitalisation and/or causing a decrease in haemoglobin level of >20 g/L and/or requiring blood transfusion that was not a haemorrhagic stroke  ^†^ ≥8 units per week ^‡^ Aspirin, clopidogrel, prasugrel, ticagrelor | |
